# Structural basis for replicase polyprotein cleavage and substrate specificity of main protease from SARS-CoV-2

**DOI:** 10.1073/pnas.2117142119

**Published:** 2022-04-05

**Authors:** Yao Zhao, Yan Zhu, Xiang Liu, Zhenming Jin, Yinkai Duan, Qi Zhang, Chengyao Wu, Lu Feng, Xiaoyu Du, Jinyi Zhao, Maolin Shao, Bing Zhang, Xiuna Yang, Lijie Wu, Xiaoyun Ji, Luke W. Guddat, Kailin Yang, Zihe Rao, Haitao Yang

**Affiliations:** ^a^Shanghai Institute for Advanced Immunochemical Studies, ShanghaiTech University, Shanghai 201210, China;; ^b^School of Life Science and Technology, ShanghaiTech University, Shanghai 201210, China;; ^c^University of Chinese Academy of Sciences, Beijing 100101, China;; ^d^State Key Laboratory of Medicinal Chemical Biology, Frontiers Science Center for Cell Response, College of Life Sciences, Nankai University, Tianjin 300384, China;; ^e^Tianjin Key Laboratory of Protein Sciences, Tianjin 300071, China;; ^f^iHuman Institute, ShanghaiTech University, Shanghai 201210, China;; ^g^The State Key Laboratory of Pharmaceutical Biotechnology, School of Life Sciences, Nanjing University, Nanjing 210023, China;; ^h^School of Chemistry and Molecular Biosciences, The University of Queensland, Brisbane, QLD 4072, Australia;; ^i^Taussig Cancer Center, Cleveland Clinic, Cleveland, OH 44195;; ^j^Laboratory of Structural Biology, School of Life Sciences, Tsinghua University, Beijing 100091, China;; ^k^Laboratory of Structural Biology, School of Medicine, Tsinghua University, Beijing 100091, China;; ^l^Shanghai Clinical Research and Trial Center, Shanghai 201210, China

**Keywords:** SARS-CoV-2, main protease, cleavage cycle, substrate selectivity

## Abstract

The main protease (M^pro^) of severe acute respiratory syndrome coronavirus 2 (SARS-CoV-2) is a key enzyme, which extensively digests CoV replicase polyproteins essential for viral replication and transcription, making it an attractive target for antiviral drug development. However, the molecular mechanism of how M^pro^ of SARS-CoV-2 digests replicase polyproteins, releasing the nonstructural proteins (nsps), and its substrate specificity remain largely unknown. Here, we determine the high-resolution structures of SARS-CoV-2 M^pro^ in its resting state, precleavage state, and postcleavage state, constituting a full cycle of substrate cleavage. The structures show the delicate conformational changes that occur during polyprotein processing. Further, we solve the structures of the SARS-CoV-2 M^pro^ mutant (H41A) in complex with six native cleavage substrates from replicase polyproteins, and demonstrate that SARS-CoV-2 M^pro^ can recognize sequences as long as 10 residues but only have special selectivity for four subsites. These structural data provide a basis to develop potent new inhibitors against SARS-CoV-2.

## Significance

COVID-19 is a deadly rampaging infectious disease with over 480 million cases worldwide. Unfortunately, effective therapies remain very limited. Novel antiviral agents are urgently needed to combat this global healthcare crisis. Here, we elucidate the structural basis for replicase polyprotein cleavage and substrate specificity of SARS-CoV-2 main protease (M^pro^). Through analyzing a series of high-resolution structures of SARS-CoV-2 M^pro^ throughout the proteolytic process, we demonstrate the molecular mechanism of M^pro^ in proteolytic processing that confers substrate specificity. Substrate selectivity is revealed using structures of the H41A mutant in complex with six individual native cleavage substrates. Our study underscores the mechanistic function of M^pro^ in the viral life cycle, which provides structural insights to develop effective inhibitors against this essential target of SARS-CoV-2.

Coronaviruses (CoVs) are prevalent pathogens that infect both humans and animals, which can lead to severe health problems and economic losses. CoVs cause a wide range of diseases, leading to damage to the respiratory, gastrointestinal, hepatic, and nervous systems (1–3). From the beginning of this century, there have been three CoVs that have crossed the species barrier and caused outbreaks of severe respiratory diseases in humans. These include severe acute respiratory syndrome (SARS) in 2002 (4), Middle East respiratory syndrome (MERS) in 2012 (5), and COVID-19 in 2019. The etiological agent for COVID-19 has been identified to be a new CoV referred to as SARS-CoV-2 (6, 7). The clinical features of COVID-19 include fever, cough, shortness of breath, and fatigue (8). SARS-CoV-2 infection can injure organs including lung, heart, liver, kidney, brain, and intestines. Older patients with baseline comorbidities are more likely to exhibit poor clinical prognosis, including death, from the infection (8). To date, there have been 480 million confirmed cases globally and more than 6.12 million deaths from this disease (9).

**Fig. 1.**
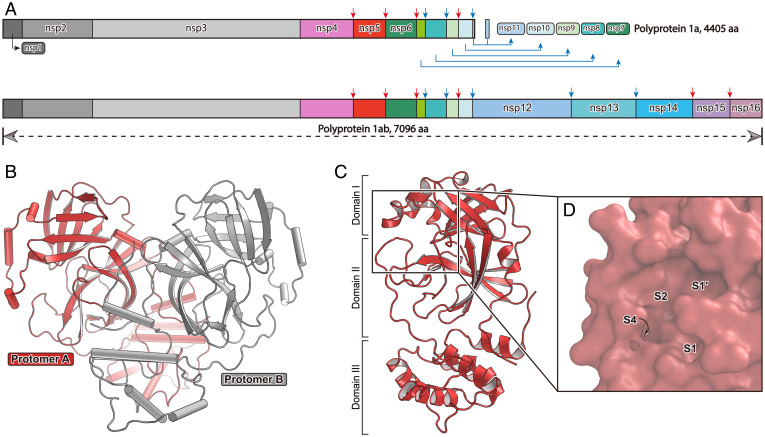
The overall structure of SARS-CoV-2 M^pro^ and its cleavage sites. (*A*) The schematic diagram of pp1a and pp1ab. The colored arrows represent the M^pro^ cleavage sites. Cleavage sites reported in this study are labeled in red. (*B*) The overall structure of the M^pro^ dimer. Protomer A and protomer B are colored in salmon and gray, respectively. (*C*) The overall structure of the M^pro^ monomer subunit. Its substrate binding pocket is shown in *D* as a surface representation. The four subsites, S1′, S1, S2, and S4, are labeled.

CoVs are positive‐sense RNA viruses with the largest viral RNA genomes ranging from 25,500 nt to 32,000 nt. The genomic length of SARS-CoV-2 is around 30,000 nt and shows a high similarity to that of SARS-CoV (6, 7). After viral entry, two overlapping polyproteins, pp1a (490 kDa) and pp1ab (794 kDa), are translated by the translational machinery of the host. These replicase polyproteins are processed into 16 nonstructural proteins (nsp1 to nsp16) including nsp12 (RNA-dependent RNA polymerase, RdRp), nsp13 (helicase), and several others, by the main protease (M^pro^, also called 3C-like serine protease/nsp5) and the papain-like protease (PL^pro^) (10, 11). M^pro^ is a cysteine protease that cleaves at no less than 11 sites on the replicase polyproteins (Fig. 1*A* and *SI Appendix*, Fig. S1 *A* and *B*) (10, 11). Initially, M^pro^ releases itself from the polyproteins through autocleavage. Then the mature M^pro^ forms a functional homodimer and transcleaves pp1a and pp1ab. However, the molecular mechanism as to how the mature SARS-CoV-2 M^pro^ cleaves pp1a and pp1ab remains largely unknown.

Given its essential role in the viral life cycle and the absence of closely related homologs in the human genome, M^pro^ is considered an attractive target for anti-CoV drug development (12–14). A program combining structure-assisted drug design, virtual drug screening, and high-throughput screening was developed to discover inhibitors with clinical potential against SARS-CoV-2 M^pro^ (15). A series of inhibitors and protease inhibitor complex structures have been reported (15–25). However, potent lead compounds with promising drug-like properties that target SARS-CoV-2 M^pro^ remain elusive to discover.

Here, we determined the high-resolution structures of SARS-CoV-2 M^pro^ in its resting state, precleavage state, and postcleavage state, providing a comprehensive view of the full catalytic cycle. Further, we solved the structures of SARS-CoV-2 M^pro^ mutant (H41A) in complex with six cleavage substrates from replicase polyproteins. Our studies provide insights into the discovery of potent inhibitors against SARS-CoV-2 M^pro^.

## Results

### Resting State of SARS-CoV M^pro^.

In order to delineate the full catalytic cycle of substrate hydrolysis for SARS-CoV-2 M^pro^, we determined the crystal structures of this peptidase in its resting state, precleavage state, and postcleavage state. We first solved the 2.0-Å apo structure of SARS-CoV-2 M^pro^, which represents its resting state. In the apo structure, there is only one protomer in an asymmetric unit, and a functional dimer is formed via *C_2_* crystal symmetry (Fig. 1*B* and *SI Appendix*, Table S1). Each protomer consists of three domains (Fig. 1*C*). The substrate binding pocket and the catalytic dyad are located at the cleft between two β-barrel fold domains (domain I and domain II) as previously described (15, 18). Domain III is relatively independent and is connected to domain II through a long loop region (residues 185 to 200). Four subsites, S1′, S1, S2, and S4, are well defined (Fig. 1*D*), and can accommodate P1′, P1, P2, and P4 positions, respectively, of the cleavage substrate. Sequence alignment shows that these subsites have preference for specific residues among the 11 cleavage sites (*SI Appendix*, Fig. S1*C*).

In the apo structure (resting state), the S1 subsite in each protomer has an absolute preference for glutamine at P1 site. This subsite is composed of the side chains of F140, N142, S144, H163, E166, and H172, and the main chain atoms of F140, L141, N142, M165, and the first residue from a neighboring protomer. The S1′ is formed by the side chains of T25, L27, H41, and C145, as well as the backbone atoms of T26 and C145. This is a shallow subsite, which can only accommodate residues with short side chains. S2 subsite is a highly hydrophobic subpocket, which consists of the side chains of H41, M49, Y54, and M165 and the alkyl portion of the side chain of Asp187. S4 is a semienclosed subsite, which is constituted by the side chains of M165, L167, F185, and Q192 and the backbone of Q189 (*SI Appendix*, Fig. S1 *D* and *E*).

### Precleavage State of SARS-CoV-2 M^pro^.

**Fig. 2.**
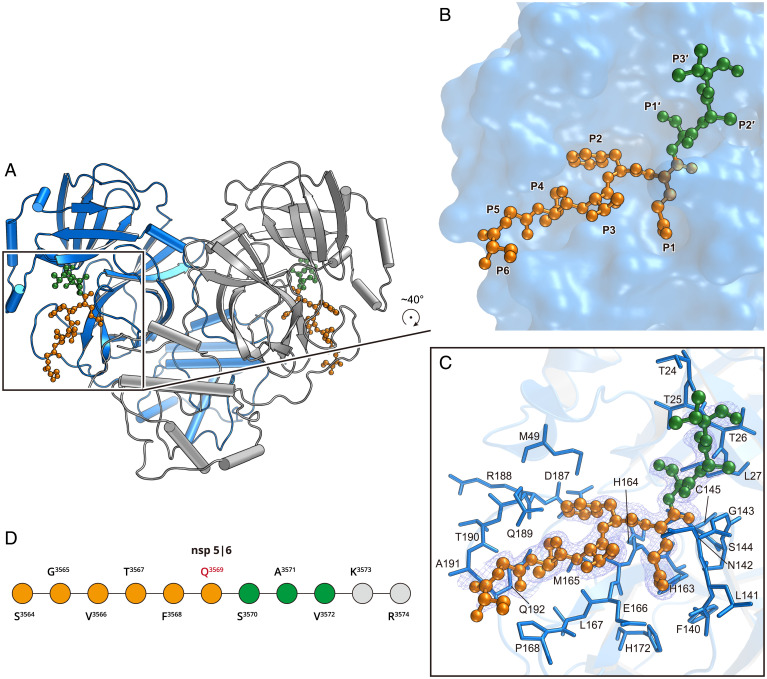
Structure of the H41A mutant in complex with the nsp5|6 peptidyl substrate. (*A*) The overall structure of H41A–nsp5|6 in dimer form. Protomer A and protomer B are colored blue and gray, respectively. (*B*) The zoom-in view of the substrate binding pocket. The nsp5|6 peptidyl substrate is shown as a ball-and-stick model. Residues from P1 to P6 and P1′ to P3′ are colored in orange and green, respectively. (*C*) The detailed interaction between M^pro^ and its cleavage substrate. Residues involved in the substrate binding are shown as marine sticks. The polder map colored as blue mesh is contoured at 2.5σ. (*D*) The schematic diagram of nsp5|6. Residues that can be traced according to the electron density map are colored in orange or green. Residues that cannot be traced are in gray.

To investigate the precleavage state of SARS-CoV-2 M^pro^, we designed an inactive mutant H41A to capture the nsp5|6 cleavage substrate (residues from S^3564^ to R^3574^ at pp1ab; *SI Appendix*, Fig. S1*B*). Then we determined the crystal structure of apo H41A at a resolution of 1.5 Å (*SI Appendix*, Fig. S2*A* and Table S1). This structure is almost identical to the resting-state structure except for its flexible C terminus (*SI Appendix*, Fig. S2*A*). Next, we solved the crystal structure of the H41A mutant in complex with the nsp5|6 substrate (H41A–nsp5|6) at 1.5 Å (Fig. 2*A*).

The complex structure is similar to that of the mutant alone with an rmsd of 0.259 Å for all Cα atoms (*SI Appendix*, Fig. S2*A*). The most significant movement can be observed for α_46–50_ and loop_188–191_ in each protomer, which is likely induced by nsp5|6 substrate binding (*SI Appendix*, Fig. S2*B*). The substrate binding pocket in the complex structure is slightly expanded compared with that in the apo mutant structure (*SI Appendix*, Fig. S2*A*). P6 to P3′ (from S^3564^ to V^3572^) can also be seen bound in the substrate binding pocket (Fig. 2 *B*–*D*). A similar structure in the precleavage state was observed in SARS-CoV M^pro^. The structure of SARS-CoV M^pro^ in the precleavage state is similar to that of SARS-CoV-2 M^pro^, with an rmsd of 0.646 Å for all Cα atoms. The major difference between these two binding modes lies in the P2', P3′, and P4' positions, as we can see for SARS-CoV-2 M^pro^ in recognizing its various peptidyl substrates. (*SI Appendix*, Fig. S3 *A* and *B*) (26).

In the S1 subsite, which is the most selective subsite, Q^3569^ at P1 position occupies this subpocket. This corresponds to the lactam ring in the Michael acceptor inhibitors reported in other complex structures (15, 18, 19). In our structure, the side chain of the glutamine is stabilized by three hydrogen bonds formed by the Oε1 atom of Q^3569^ and Nε2 atom of H163, the Nε2 atom of Q^3569^ and carbonyl oxygen of F140, and the Nε2 atom of Q^3569^ and an ordered water molecule (*SI Appendix*, Fig. S2*C*). These three hydrogen bonds impose an absolute requirement for glutamine in the S1 subsite. The main chain carbonyl oxygen of Q^3569^ occupies the oxyanion hole, which is stabilized by the amide groups of G143 and C145 (*SI Appendix*, Fig. S2*C*).

The S2 subsite is a deep hydrophobic pocket, which is slightly expanded to accommodate the bulky side chain of F^3568^ at the P2 site compared with the apo mutant structure. M49, M165, and the alkyl portion of D187, R188, and Q189 are involved in hydrophobic interactions (Fig. 2*C*). S3 is solvent exposed so that it can tolerate a wide range of functional groups at P3 site. Two hydrogen bonds formed by the amide group of T^3567^ at P3 with the carbonyl oxygen of E166 and the carbonyl oxygen of T^3567^ with the amide group of E166 stabilized the substrate (*SI Appendix*, Fig. S2*C*). S4 is occupied by a small residue, V^3566^ (Fig. 2 *B*–*D*). Hydrophobic interaction between the side chain of V^3566^ and the side chains of M165, L167, and Q192 contributes to substrate recognition (Fig. 2*C* and *SI Appendix*, Fig. S2*B*). It is interesting to note that Q189 is not only involved in S2 subsite formation but also participates in stabilization of the S4 subsite. Due to the steric hindrance of the benzyl group of F^3568^ at the P2 site, Q189 moves outward to interact with the main chain at the P4 site (*SI Appendix*, Fig. S2 *B* and *C*). Two pairs of hydrogen bonds are formed by the amide group of V^3566^ and the Oε1 atom of Q189, and by the carbonyl oxygen of V^3566^ and the Nε2 atom of Q189 (*SI Appendix*, Fig. S2*C*). It is observed that α_46–50_ and loop_188–191_ move outward from the binding site (*SI Appendix*, Fig. S2 *A* and *B*).The P5 and P6 sites are partially exposed to the solvent, and interact with P168 and A191 of the protease through van der Waals interactions (Fig. 2*C* and *SI Appendix*, Fig. S2*B*).

S1′ is a shallow subsite, which can only hold small residues such as S^3570^ at P1′ (Fig. 2*C*). Here, the carbonyl oxygen of S^3570^ and the amide group of G143 form a hydrogen bond (*SI Appendix*, Fig. S2*C*). The side chains of T25, L27, and C145 interact with S^3570^ via van der Waals interactions. At the S2′ subsite, the main chain of A^3571^ at P2′ is stabilized by two hydrogen bonds, which are formed between the amide group of A^3571^ and the carbonyl oxygen of T26, and between the carbonyl oxygen of A^3571^ and the amide group of T26 (*SI Appendix*, Fig. S2*C*). The side chain of A^3571^ is exposed to solvent, so it does not contribute significantly to substrate binding (Fig. 2 *B* and *C*). At the S3′ subsite, V^3572^ is at P3′ and is also solvent exposed. The S3′ subsite shows low sequence conservation among 11 cleavage sites (*SI Appendix*, Fig. S1*B*). It only forms weak van der Waals interaction with T24 (Fig. 2*C* and *SI Appendix*, Fig. S2*B*). The last two residues of the substrate cannot be traced, due to the poor electron density in this region.

### Postcleavage State of SARS-CoV-2 M^pro^.

**Fig. 3.**
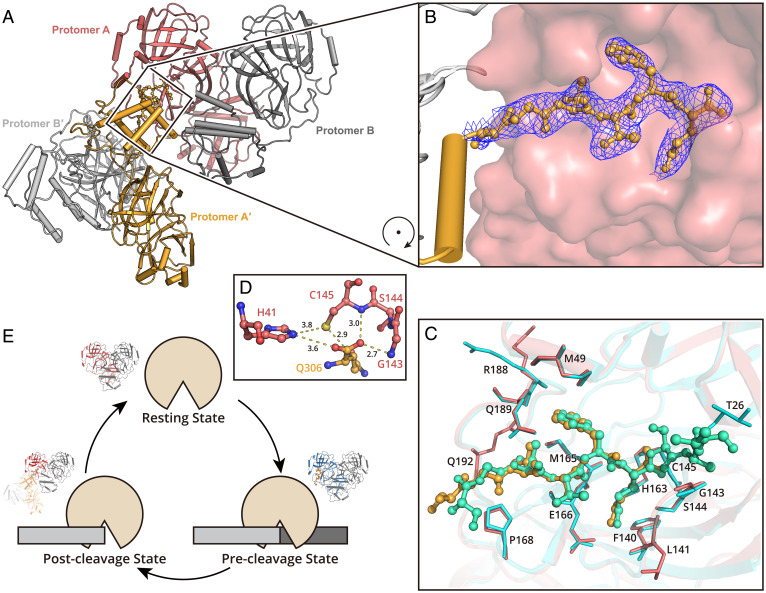
Structure of SARS-CoV-2 M^pro^ in the postcleavage state. (*A*) Two M^pro^ dimers are associated together, representing the postcleavage state. One dimer pair is colored in salmon (protomer A) and gray (protomer B). A second dimer is formed by protomer A′ and B′ (bright orange and light gray). The C terminus of protomer A′ is inserted into the substrate binding pocket of protomer A. (*B*) The zoom-in view of the C terminus of protomer A′. The residues S301 to Q306 are shown as a ball-and-stick model. The 2*F_o_*–*F_c_* density map contoured at 1.0σ is shown in blue mesh. (*C*) A comparison of the H41A–nsp5|6 complex structure and M^pro^ in postcleavage state structure. The H41A–nsp5|6 complex is colored in cyan and green (protease in cyan and peptidyl substrate nsp5|6 in green), and M^pro^ in postcleavage state is colored in salmon and bright orange (protease in salmon and C-terminal protomer A′ in bright orange). Substrates located in substrate binding picket are shown as ball-and-stick models. Residues involved in substrate binding are shown as sticks. (*D*) The arrangement of amino acids in the catalytic dyad. (*E*) A cartoon visualization of the cleavage cycle of SARS-CoV-2 M^pro^.

In an attempt to crystallize wild-type (WT) apo M^pro^, we obtained a crystal form of the enzyme that belongs to the *P*_1_ space group (*SI Appendix*, Table S1). We determined this crystal structure at 2.2 Å, and it showed there are two pairs of M^pro^ dimers (AB and A′B′) in an asymmetric unit (Fig. 3*A*). The AB dimer is almost identical to the A′B′ dimer, with an rmsd of 0.065 Å for all Cα atoms. Interestingly, the C-terminal residues S301 to Q306 (corresponding to S^3564^ to Q^3569^ in the polyprotein) from the neighboring protomer A′ in the A′B′ dimer are observed to fill the substrate binding pocket of protomer A in the AB dimer (Fig. 3 *A* and *B*). Similar conformation was observed in SARS-CoV M^pro^, suggesting this is an important intermediate state for these two viral proteases in the cleavage cycle (*SI Appendix*, Fig. S3 *C* and *D*) (27). This structure therefore represents the postcleavage state for SARS-CoV-2 M^pro^ in the processing of the nsp5|6 substrate, where the P1′ to P4′ portion has already been released. The P1 to P6 sites take on conformations similar to those in the precleavage state (Fig. 3*C*). At the active center, the complete carboxyl terminus of Q^3569^ (P1 site) is close to the thiol group of the C145 nucleophile whose thiol sulfur is 3.8 Å from the Nε2 of the base H41 (Fig. 3*D*). Thus, it is possible to envisage the full cycle of substrate hydrolysis (Fig. 3*E*). Additional interactions between protomer A′ and protomer B (*SI Appendix*, Fig. S4) are also observed which may play a role in the cleavage event.

It has been observed that domain III of each protomer is flexible (*SI Appendix*, Fig. S5*A*), and the most flexible region is located at its C terminus (*SI Appendix*, Fig. S5*B*). The alignment of protomer A and protomer B shows that the loop connecting domains I and II serves as a hinge, which allows domain III to rotate by ∼5° (*SI Appendix*, Fig. S5*C*). These conformational changes may be important in facilitating efficient substrate recognition and catalysis.

### Substrate Specificity of SARS-CoV-2 M^pro^.

**Fig. 4.**
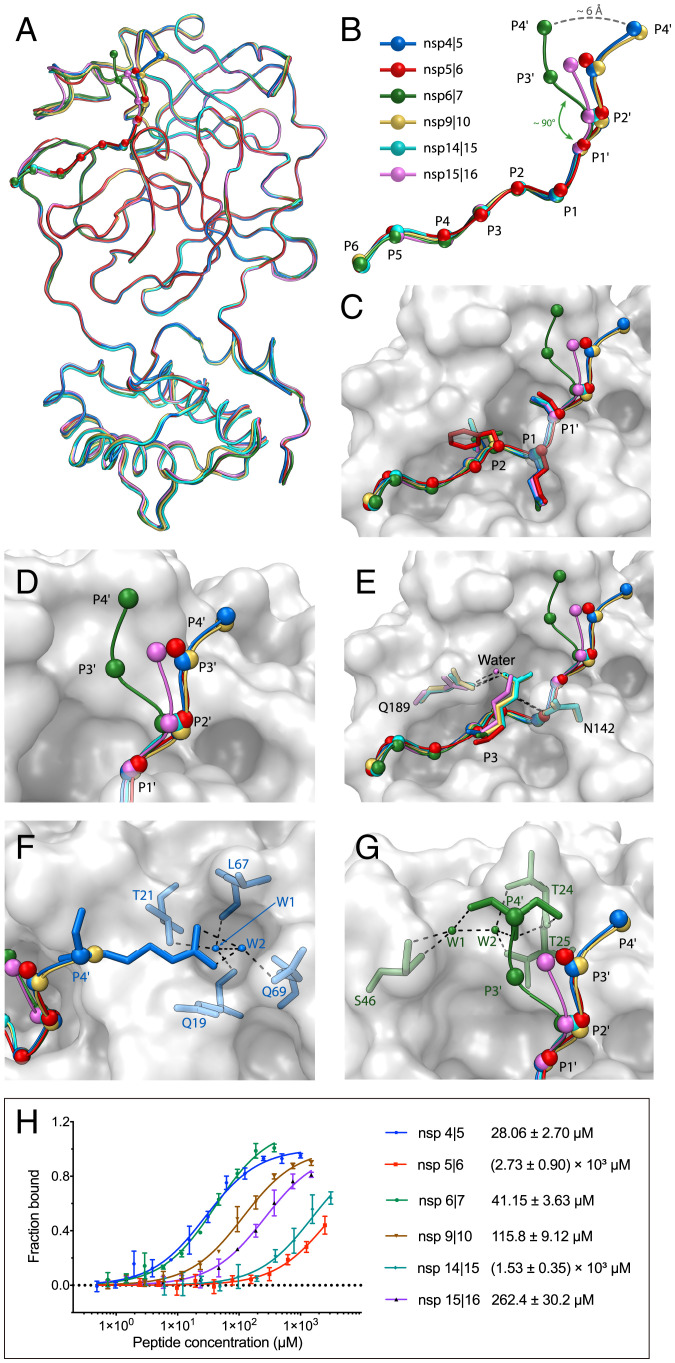
Structure of H41A mutant in complex with peptidyl substrates. (*A*) A comparison of the structures of six peptidyl substrates in complex with the H41A mutant. The structures of H41A–nsp4|5, H41A–nsp5|6, H41A–nsp6|7, H41A–nsp9|10, H41A–nsp14|15, and H41A–nsp15|16 are colored light blue, bright red, dark green, yellow orange, bright cyan, and bright purple, respectively. (*B*) Overlay of Cαs from each substrate. The Cα of each residue is shown as a colored sphere. (*C*) A zoom-in view of the substrate binding pocket with six peptidyl substrates. The side chain of residues at the most highly conserved subsites (S1, S2, and S1′) are shown in stick models. (*D*) Cα positions are divergent from P2′ to P4′ sites. (*E*) Positively charged residues at P3 position make extra interactions with H41A. The interacting residues are shown as stick models. Water molecule is shown as a magenta sphere. (*F*) R^3267^ at P4′ position in the nsp4|5 substrate makes additional interactions with H41A. The residues that interact are shown as sticks. Water molecules are shown as blue spheres and named W1 and W2, respectively. (*G*) S^3863^ at P4′ position in the nsp6|7 substrate makes additional interactions with M^pro^. The interacting residues are shown as stick models. Water molecules are shown as green spheres and named W1 and W2, respectively. (*H*) MST assay curve of the binding affinity between H41A and six peptidyl substrates. Data from three independent experiments are presented as the mean values with their SD.

**Fig. 5.**
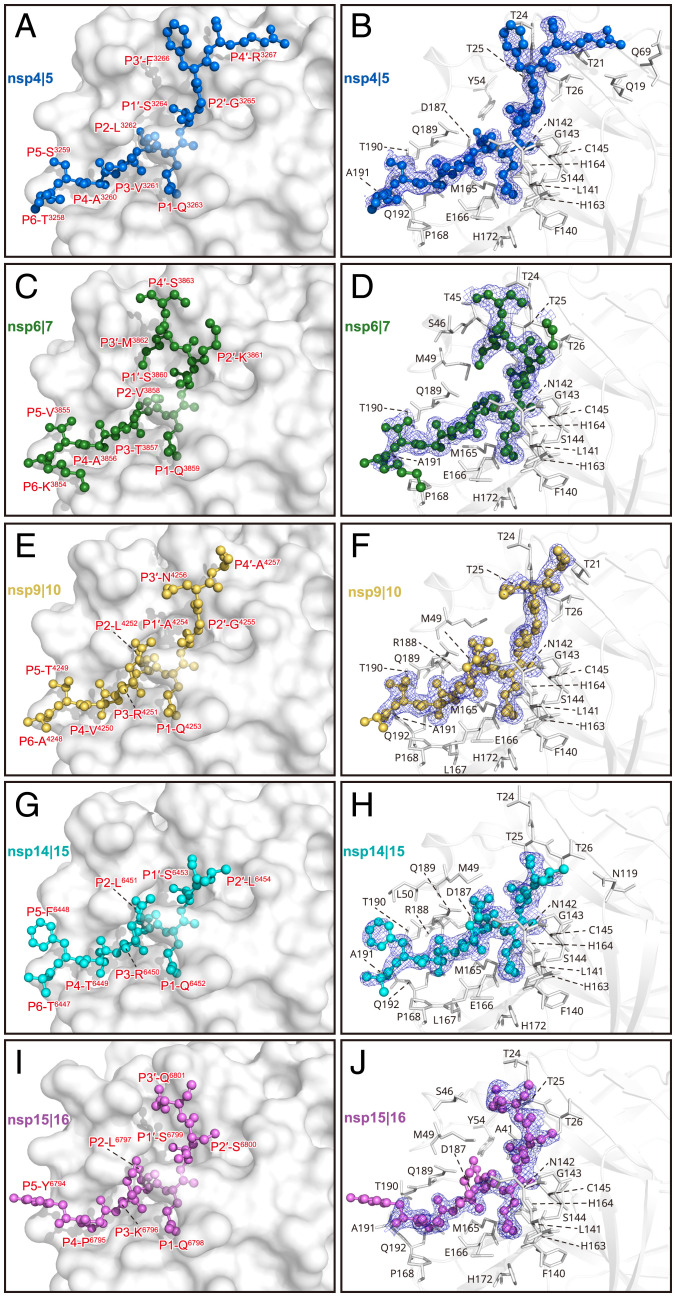
The binding modes of the peptides to the H41A mutant: (*A* and *B*) nsp4|5, (*C* and *D*) nsp6|7, (*E* and *F*) nsp9|10, (*G* and *H*) nsp14|15, and (*I* and *J*) nsp15|16. The cleavage substrates are shown as balls and sticks. The residues that participate in the substrate binding are shown as sticks. The polder maps are colored in blue mesh and contoured at 2.5σ.

In order to understand the substrate preference of M^pro^ in processing the polyproteins, we determined the structure of M^pro^ mutant H41A in complex with a series of substrates derived from multiple cleavage sites on polyproteins. In addition to the aforementioned crystal structure of H41A–nsp5|6 peptide, we successfully solved the structures of H41A complexed with nsp4|5, nsp6|7, nsp9|10, nsp14|15, and nsp15|16 substrates, respectively (Figs. 4*A* and 5 and *SI Appendix*, Fig. S6 and Table S1). The overall structure is almost identical in all six complex structures, with rmsd values from 0.118 Å to 0.324 Å for all Cα atoms (Fig. 4*A*). Interestingly, superposition of these structures shows that the Cα positions of P6 to P1′ for different peptidyl substrates are well aligned, indicating that SARS-CoV-2 M^pro^ has strong selectivity at these cleavage sites (Fig. 4 *B* and *C*). In contrast, Cα positions from P2′ to P4′ sites are sharply divergent (Fig. 4*D*), which suggests that S2′ to S4′ subsites in SARS-CoV-2 M^pro^ would not have a stringent preference on substrate side chains, reflecting their higher degree of plasticity compared with S6 to S1′ subsites.

A closer look at the subsites in these six H41A–substrate complex structures reveals that S1 is only occupied by glutamine because the side chain of P1 glutamine can form three hydrogen bonds with the side chain of H163, the main chain of F140, and an ordered water molecule, respectively (Fig. 5 and *SI Appendix*, Fig. S7). The S2 subsite is a deep hydrophobic subpocket which prefers hydrophobic residues, and hydrophobic interactions dominate the interplay between the protease and the substrate at this subsite. This subsite is highly malleable and can tolerate the side chains from a range of hydrophobic residues, such as leucine, valine, and phenylalanine. Leucine appears at P2 position in 9 out of 11 peptidyl substrates (*SI Appendix*, Fig. S1*B*), indicating that it is the most preferred residue, as its slim isobutyl-containing side chain may more favorably insert into this deep subsite. This can be seen in the H41A–nsp4|5, H41A–nsp9|10, H41A–nsp14|15, and H41A–nsp15|16 complex structures. In contrast, valine (in the nsp6|7 complex structure), with a smaller side chain, cannot reach to the bottom of S2. Distinct from the nsp5|6 complex structure, where Q189 moves outward to interact with the main chain of P4 site due to the steric hindrance as mentioned above (*SI Appendix*, Fig. S2*B*), in the other five complex structures, Q189 participates in positioning these five substrates through a hydrogen bond formed by its own Oε1 atom and the amine moiety of the amide group at all these P2 sites (*SI Appendix*, Fig. S7).

It has been assumed in the literature that the solvent-accessible S3 is not selective (15, 18), so it has not been considered to play a significant role in drug development. Among all cleavage sites on the replicase polyproteins, this position can be occupied by five different residues, including hydrophobic residues (valine and methionine), polar residue (threonine), and positively charged residues (lysine and arginine) (*SI Appendix*, Fig. S1*B*). In the complex structures we have determined, there are four residues that appear at this position (valine in nsp4|5, threonine in nsp5|6 and nsp6|7, arginine in nsp9|10 and nsp14|15, and lysine in nsp15|16). All the main chains are positioned by two hydrogen bonds as has been described in the H41A–nsp5|6 complex structure (*SI Appendix*, Fig. S7). It is notable that the side chains of positively charged residues (arginine or lysine) at the P3 position are able to form hydrogen bonds with H41A, thus contributing to substrate binding. In the structure of H41A in complex with nsp9|10, the side chain of R^4521^ at P3 forms two hydrogen bonds with Q189 and N142 (Fig. 4*E* and *SI Appendix*, Fig. S7*C*); in the structure of H41A in complex with nsp15|16, the side chain of K^6796^ at the corresponding site forms a hydrogen bond with a surrounding water molecule which bridges both K^6796^ from the substrate and Q189 from the protease (Fig. 4*E* and *SI Appendix*, Fig. S7*E*). These findings suggest that a suitable functional group at the P3 site can be created to assist in increasing inhibitor potency and selectivity. Compared with the S3 subsite that can tolerate a variety of residues, S4 is a small subpocket which can only accommodate residues with small side chains. In the complex structures, four different residues with small side chains (alanine, valine, proline, and threonine) can be seen to bind to this subsite (*SI Appendix*, Fig. S1*B*). Compared with the H41A–nsp5|6 complex structure, where Q189 interacts with the main chain of the P4 site, only the carbonyl oxygen of T190 is involved in the substrate positioning by forming a hydrogen bond with the amide nitrogen group from the residue at the P4 site in H41A–nsp4|5, H41A–nsp6|7, H41A–nsp9|10, and H41A–nsp14|15 complex structures (*SI Appendix*, Figs. S2*C* and S7 *A*–*D*). Interestingly, T^6449^ at P4 forms four hydrogen bonds with T190 and R188 in the complex with nsp14|15 (*SI Appendix*, Fig. S7*D*). Here, the Oγ from the side chain of this threonine interacts with the carbonyl oxygen of R188, the amide nitrogen group of T190, and the carbonyl oxygen of T190. Similar to the structure of H41A–nsp5|6 peptide, residues at the P5 and P6 sites are solvent exposed in the other five complex structures, thus having limited selectivity. In these structures, only the carbonyl oxygen from the P5 position can form a hydrogen bond with the protease through a bridging water molecule (H41A–nsp4|5, H41A–nsp6|7, and H41A–nsp9|10). The side chain of S^3259^ also forms a hydrogen bond with the carbonyl oxygen of Q189 through a bridging water molecule in the H41A–nsp4|5 complex structure (*SI Appendix*, Fig. S7*A*). Except for F^6793^ at the P6 position that cannot be traced in the structure of H41A–nsp15|16 complex, the residues at this position in other complexes are visible (Fig. 4*B* and *SI Appendix*, Fig. S6).

As S1′ is a shallow pocket, it prefers small residues. All six structures show that serine (in nsp4|5, nsp5|6, nsp6|7, nsp14|15, and nsp15|16) and alanine (in nsp9|10) can be accommodated at this subsite, while serine is the most common residue appearing at the P1′ position in the substrate (*SI Appendix*, Fig. S1*B*). A water molecule bridges the side chain of serine from the substrate to the main chain of A41 through hydrogen bonds, which may account for serine being frequently observed at this site (*SI Appendix*, Fig. S7 *A*, *B*, *D*, and *E*). The side chain at P1′ also interacts with the side chains of T25, L27, and C145 through van der Waals interactions. The backbone atoms of P2′ are confined to a relatively fixed position through two hydrogen bonds formed by the amide group of P2′ with the carbonyl oxygen of T26 and the carbonyl oxygen of P2′ residue with the amide group of T26 (*SI Appendix*, Fig. S7). The side chains of P2′ are also exposed to the solvent, and thus S2′ is not expected to have a strong substrate selectivity. Only in the H41A–nsp15|16 complex structure, the side chain of S^6800^ at P2′ position forms hydrogen bonds with N119 and G143 through water molecules (*SI Appendix*, Fig. S7*E*).

The positions for the Cαs become diverse beyond the P2′ site (i.e., out to P3′ and P4'). The directions of the backbones for these substrates diverge into two different directions at the P3′ position, noting that, in the H41A–nsp14|15 complex, the backbone can only be traced to P2′ (Figs. 4*B* and 5 *G* and *H*). In the structures for nsp4|5, nsp5|6, nsp9|10, and nsp15|16, their backbone extension directions share a common feature. In contrast, in the structure of H41A–nsp6|7 complex, M^3862^ at P3′ has a sharp turn of nearly 90°, causing the deviation of its backbone direction from those in the other complexes (Fig. 4 *B* and *D*). Only in the H41A–nsp15|16 complex, the density for the side chain of Q^6801^ at P3′ can be observed (Fig. 5 *I* and *J*). In this structure, the main chain carbonyl oxygen 15|16 forms a hydrogen bond with a water molecule, which takes on the bridge to T24 and T26. In addition, the Nε2 from its side chain makes a hydrogen bond with T25 Oγ (*SI Appendix*, Fig. S7*E*).

The density can be traced to P4′ merely in three substrate complex structures (H41A–nsp4|5, H41A–nsp6|7, and H41A–nsp9|10) (*SI Appendix*, Fig. S6). In the H41A–nsp4|5 complex, the main chain NH group of R^3267^ interacts with T24 through a hydrogen bond. Two water molecules form a bridge with the side chains of Q19, T21, L67, and Q69 through hydrogen bonds (Figs. 4*F* and 5 *A* and *B* and *SI Appendix*, Fig. S7*A*). In the H41A–nsp9|10 structure, only one hydrogen bond is formed between the main chain NH group of A^4257^ and T24 (*SI Appendix*, Fig. S7*C*). The Cα position of S^3863^ at the P4′ position in the nsp6|7 substrate is 6 Å from those at the P4′ site in the other two complex structures (Fig. 4*B*). An ordered water molecule forms hydrogen bonds with the main chain NH group of S^3863^, T24, and T25 (Figs. 4*G* and 5 *C* and *D* and *SI Appendix*, Fig. S7*B*). Another water molecule forms hydrogen bonds with the main chain carbonyl group of S^3863^ and S46 (Figs. 4*G* and 5 *C* and *D* and *SI Appendix*, Fig. S7*B*).

We next measured the binding affinity between the H41A mutant and the six substrates. The *K_d_* values ranges from 28.1 μM to 2.7 mM (Fig. 4*H*). Nsp4|5, nsp6|7, and nsp9|10 have the strongest binding affinity for H41A, with *K_d_* values of 28.1 μM, 41.2 μM, and 115.8 μM, respectively. This is consistent with the fact that these substrate backbones can be traced to as long as 10 residues (P6 to P4′) in the density maps. Two other cleavage substrates (nsp14|15 and nsp15|16) have lower binding affinity, with *K_d_* values of 1.5 mM and 262.4 μM. In these two complexes, eight residues can be seen, including the P5 to P3′ sites for nsp14|15 and the P6 to P2′ sites for nsp15|16. However, serine at P2′ and glutamine at P3′ in the nsp15|16 substrate form three extra hydrogen bonds compared with the nsp14|15 substrate complex. Hence, the nsp15|16 cleavage substrate is able to form stronger binding to H41A than the nsp14|15 substrate. Although the backbones of P6 to P3′ can be traced in the nsp5|6 substrate complex structure, there are fewer hydrogen bond interactions than in the other five structures, and it is reasonable that it has the lowest binding affinity to H41A, with a *K_d_* value of 2.7 mM. The discrepancy of the binding affinity between the cleavage substrates and H41A suggests that viral protease might have a preference to release certain nsps from the polyprotein in the event of replicase assembly, which can be taken advantage of to design more potent inhibitors.

## Discussion

New drugs are urgently needed to treat SARS-CoV-2 infections. Significant efforts have been focused on targeting the evolutionarily conserved nonstructural proteins, which are involved in viral replication and transcription (14, 15, 28–30). Among them, M^pro^ is one of the most attractive targets, due to its pivotal role in replicase polyprotein cleavage and the fact that it is evolutionarily conserved across all CoVs (12, 13). Highly selective inhibitors against M^pro^ could be potential broad-spectrum drug candidates to fight against CoV infections not only for today but also in the future (12). One step toward the rational design of potent and selective inhibitors that target M^pro^ can be achieved by discovering the detailed mechanisms for the process of proteolysis and substrate recognition. In this work, we determined the structures of viral protease at the resting state, precleavage state, and postcleavage state (Fig. 3*E*). Our findings showed that domain III of M^pro^ is the most flexible domain. It can adjust its conformation to allow its C terminus to access the substrate binding pockets from its surrounding active M^pro^s (*SI Appendix*, Fig. S5*A*). The most flexible region lies at C terminus (S301 to Q306) of M^pro^, which has three preferred orientations (*SI Appendix*, Fig. S5*B*). The flexibility of this region is important for regulation of its C-terminal autocleavage process. Particularly, the M^pro^ C terminus in orientation 2 may allow itself to favorably insert into the substrate binding pocket of its neighbor M^pro^ during C-terminal proteolysis. This suggests that small molecules which can block the conformational change of domain III may serve as allosteric inhibitors for M^pro^ (31).

Although many screened or designed inhibitors that target the substrate binding pocket of M^pro^ have been discovered or developed (15, 17, 18), potent inhibitors with clinical potential are still needed. In this study, we have solved the crystal structures of M^pro^ mutant H41A in complex with six cleavage substrates, respectively. These structures reveal that SARS-CoV-2 M^pro^ can recognize substrates as long as 10 residues but generally have special selectivity for four subsites (S1, S1′, S2, and S4). In addition to the most conserved S1 subsite, the highly plastic S2 subsite is also important for drug design. The S2 subsite is a deep hydrophobic pocket which prefers hydrophobic residues. Interestingly, this subsite is highly malleable, a factor that can be taken into account for drug development. Although it has been assumed that P3 does not have selectivity, in the structures of H41A–nsp9|10, H41A–nsp14|15, and H41A–nsp15|16 complexes, arginine or lysine at P3 can form hydrogen bonds with the protease through their long side chains (Fig. 5 *F*, *H*, and *J*). Thus, the P3 position can serve as a subsite to enhance protease inhibitor binding. These structures also indicate that the P2′ to P4′ sites demonstrate a higher conformational diversity than those for the P6 to P1′ sites, due to the lack of protease substrate specificity at these positions. We compared several published M^pro^–inhibitor complex structures (15, 16, 18, 19, 21, 32–34) with the structure of M^pro^ in complex with nsp4|5, which has the strongest binding affinity to M^pro^ among the substrates we tested (*SI Appendix*, Fig. S8). N3, 11a, 11b, 13b, boceprevir, and nirmatrelvir (PF-07321332) are peptidomimetic inhibitors which mainly target S1, S1′, S2, and S4 sites. All of them form a covalent bond to C145, although the structure of each warhead can be different. N3 occupies S1, S2, and S4 and partially occupies S1′ (*SI Appendix*, Fig. S8*A*). Inhibitors 11a and 11b occupy S1, S2, and S4 (*SI Appendix*, Fig. S8 *B* and *C*). The cyclohexyl moiety of 11a and the 3-fluorophenyl group of 11b at P2 sites can both be snugly accommodated by the bulky S2 subsite. Inhibitor 13b is bound to the viral protease in a similar mode to 11a and 11b, but its Boc group does not occupy the S4 subsite (*SI Appendix*, Fig. S8*D*). This is the likely explanation for the lower potency of 13b. In the M^pro^–boceprevir complex structure, although cyclobutylalanice at the P1 site can fit in the S1 subsite, it does not form as strong an interaction as other inhibitors (*SI Appendix*, Fig. S8*E*). In contrast, nirmatrelvir (PF-07321332) is a new oral clinical drug that shares a similar rigid dimethylcyclopropylproline group of its P2 site but has a classical (S)-γ-lactam ring at the P1 site, and demonstrates a strong potency as an oral therapeutic for COVID-19 (*SI Appendix*, Fig. S8*F*) (33, 34). Compound 23 is a perampanel-derived noncovalent inhibitor that binds to S1, S1′, S2, and S4 sites and demonstrates promising antiviral activity (*SI Appendix*, Fig. S8*G*) (32). Pelitinib, which exhibits a strong antiviral activity, binds to a new allosteric site close to the most flexible region (S301 to Q306) in domain III and hampers the movement of the C-terminal tail (*SI Appendix*, Fig. S8*H*) (31).

In summary, our comprehensive structural studies provide valuable insights for drug design. We demonstrate that the subpockets (S1, S1′, S2, and S4) which are endowed with an exceptional selectivity are important for selective and potent binding. Additionally, other subsites such as S3 and S2′ to S4′, which have less specificity for substrate recognition, may also be used to enhance inhibitor binding and to specifically target M^pro^.

## Materials and Methods

### Cloning, Protein Expression, and Purification of COVID-19 Virus M^pro^.

The cell cultures were grown and the protein was expressed according to a previous report (15). The cell pellets were resuspended in lysis buffer (20 mM Tris⋅HCl pH 8.0, 150 mM NaCl, 5% glycerol), lysed by high-pressure homogenization, and then centrifuged at 25,000 × *g* for 30 min. The supernatant was loaded onto a nickel-nitrilotriacetic acid (Ni-NTA) affinity column (Qiagen), and washed in lysis buffer containing 20 mM imidazole. The His-tagged M^pro^ was eluted using lysis buffer containing 300 mM imidazole. The imidazole was then removed through desalting. Human rhinovirus 3C protease was added to remove the C-terminal His tag. SARS-CoV-2 M^pro^ was further purified by ion exchange chromatography. The purified M^pro^ was transferred to 10 mM Tris⋅HCl pH 8.0 by desalting and stored at −80 °C until needed.

The H41A construct design was based on the WT M^pro^ reported earlier (15). It was then cloned into a modified pET-32a vector with an N-terminal thioredoxin tag and a SARS-CoV M^pro^ cleavage site. The vector was transferred into BL21 (DE3) and induced with isopropyl β-D-thiogalactoside for protein expression. The affinity purification was the same as for WT. The elution solution was concentrated, and imidazole was removed through desalting. Next, the thioredoxin tag was cleaved by SARS-CoV M^pro^ overnight at 4 °C. Next, a Ni^2+^ affinity purification step was used to remove thioredoxin tag and SARS-CoV M^pro^. The eluant was then loaded onto a desalting column to remove imidazole. The eluant was collected and cleaved by human rhinovirus 3C protease overnight at 19 °C. Finally, another Ni^2+^ affinity purification step was used to separate His tag cleaved and noncleaved protein. The flow-through sample was concentrated, and further purified by ion exchange chromatography. Then the purified protein was transferred to 10 mM Tris⋅HCl pH 8.0.

### Crystallization, Data Collection, and Structure Determination.

SARS-CoV-2 M^pro^ was concentrated to 5 mg/mL and mixed in a 1:1 (vol/vol) ratio with the well buffer, then crystallized by the hanging drop vapor diffusion method at 20 °C. The best crystals were grown using a well buffer containing 0.1 M imidazole pH 8.1, 10% polyethylene glycol (PEG) 3000, 0.2 M Li_2_SO_4_. The cryoprotectant solution was the same as the reservoir but with 20% glycerol added.

The procedure for the SARS-CoV-2 M^pro^ H41A mutant was the same as for the WT M^pro^, except the best crystals were grown using a well buffer containing 0.1 M MES (pH 5.4 to 5.8), 7% PEG 6000, and 6% dimethyl sulfoxide (DMSO). The cryoprotectant solution was the same as the reservoir but with 20% glycerol added.

The synthesized peptides (GL Biochem) were dissolved in a well buffer (0.01 M MES pH 5.6, 3.5% PEG 6000, and 3% DMSO) at 10 mM. Next, the dissolved peptide was added to the drop that contained crystals in a 1:1 (vol/vol) ratio and soaked for 12 h. The cryoprotectant solution was the same as for the M^pro^ H41A mutant.

X-ray data were collected on beamlines BL17U1 and BL19U1, Shanghai Synchrotron Radiation Facility (SSRF) at 100 K using an Eiger X 16M image plate detector or Pilatus3 6M image plate detector. Data integration and scaling were performed using the programs XDS (35) and xia2 (36). The apo structure was determined by molecular replacement (MR) with PHASER (37), a program inside the Phenix 1.17.1 package (38). The COVID-19 virus M^pro^ (Protein Data Bank [PDB] ID: 6LU7) was used as a search template. The model from MR was subsequently subjected to iterative cycles of manual model adjustment with Coot 0.8 (39), and refinement was completed with Phenix REFINE (40). The substrates were built according to the omit map. The phasing and refinement statistics are summarized in *SI Appendix*, Table S1.

### Microscale Thermophoresis Assay.

The microscale thermophoresis (MST) assays were carried out according to the method previously reported (41). Binding affinities of peptidyl substrates with SARS-CoV-2 M^pro^ (H41A) were measured using Monolith NT.Automated (NanoTemper Technologies). H41A was fluorescently labeled according to the manufacturer’s procedure. Protein was kept in MST Buffer (20 mM Hepes pH 7.0, 300 mM NaCl, 5% glycerol), and the concentration was adjusted to 4 μM. Next, RED-Tris-NTA second-generation dye was added, mixed, and incubated for 30 min at 25 °C in the dark. The final molar ratio of protein to dye was 80:1. Each of the unlabeled peptidyl substrates at 12 different serially diluted concentrations was then mixed with the same volume of labeled protein at room temperature. The samples were then loaded into standard treated capillaries (NanoTemper Technologies) and measured at 25 °C, 40% LED power, and medium MST power. Each assay was repeated three times. Data analyses were performed using MO.Affinity Analysis v.2.2.4 software (NanoTemper Technologies). All of the final plots were made using GraphPad Prism 8.0.

## Supplementary Material

Supplementary File

## Data Availability

All study data are included in the article and/or *SI Appendix*. The coordinates and structure factors have been deposited in the PDB, under the following ID codes: 6M03 for M^pro^-Apo, 7VAH for H41A, 7E5X for M^pro^-Post-cleavage state, 7DVP for H41A-nsp4|5, 7DVW for H41A-nsp5|6, 7DVX for H41A-nsp6|7, 7DVY for H41A-nsp9|10, 7DW6 for H41A-nsp14|15, 7DW0 for H41A-nsp15|16.
