# Supplementary material for: Structural basis for replicase polyprotein cleavage and substrate specificity of main protease from SARS-CoV-2
Source: Proc Natl Acad Sci U S A. 2022 Apr 5;119(16):e2117142119. doi: 10.1073/pnas.2117142119 (PMC9172370; doi:10.1073/pnas.2117142119)
Supplement: Supplementary File [file pnas.2117142119.sapp.pdf]

Supplementary Information for

Structural basis for replicase polyprotein cleavage and substrate specificity of main protease from SARS-CoV- 2

Yao Zhao[a,b,1,2], Yan Zhu[a,b,c1], Xiang Liu[d,e,1], Zhenming Jin[a,b], Yinkai Duan[a,b], Qi Zhang[a,b], Chengyao Wu[a,b], Lu Feng[a,b], Xiaoyu Du[a,b], Jinyi Zhao[a,b], Maolin Shao[a,b], Bing Zhang[a,b], Xiuna Yang[a,b], Lijie Wu[f], Xiaoyun Ji[g], Luke W. Guddat[h], Kailin Yang[i], Zihe Rao[a,b,d,e,j,k,2], Haitao Yang[a,b,l,2]

[a]Shanghai Institute for Advanced Immunochemical Studies, ShanghaiTech University, Shanghai 201210, China;
[b]School of Life Science and Technology, ShanghaiTech University, Shanghai 201210, China;
[c]University of Chinese Academy of Sciences, Beijing 100101, China;
[d]State Key Laboratory of Medicinal Chemical Biology, Frontiers Science Center for Cell Response, College of Life Sciences, Nankai University, Tianjin 300384, China;
[e]Tianjin Key Laboratory of Protein Sciences, Tianjin 300071, China;
[f]iHuman Institute, ShanghaiTech University, Shanghai 201210, China;
[g]The State Key Laboratory of Pharmaceutical Biotechnology, School of Life Sciences, Nanjing University, Nanjing 210023, China;
[h]School of Chemistry and Molecular Biosciences, The University of Queensland, Brisbane, QLD 4072, Australia;
[i]Taussig Cancer Center, Cleveland Clinic, Cleveland, OH 44195;
[j]Laboratory of Structural Biology, School of Life Sciences, Tsinghua University, Beijing 100091, China;
[k]Laboratory of Structural Biology, School of Medicine, Tsinghua University, Beijing 100091, China;
[l]Shanghai Clinical Research and Trial Center, Shanghai 201210, China

[1]Y.Z., Yan.Z. and X.L. contributed equally to this work.
[2]To whom correspondence may be addressed. Email: zhaoyao@shanghaitech.edu.cn; raozh@mail.tsinghua.edu.cn; yanght@shanghaitech.edu.cn

This PDF file includes:

Figures S1 to S8
Tables S1

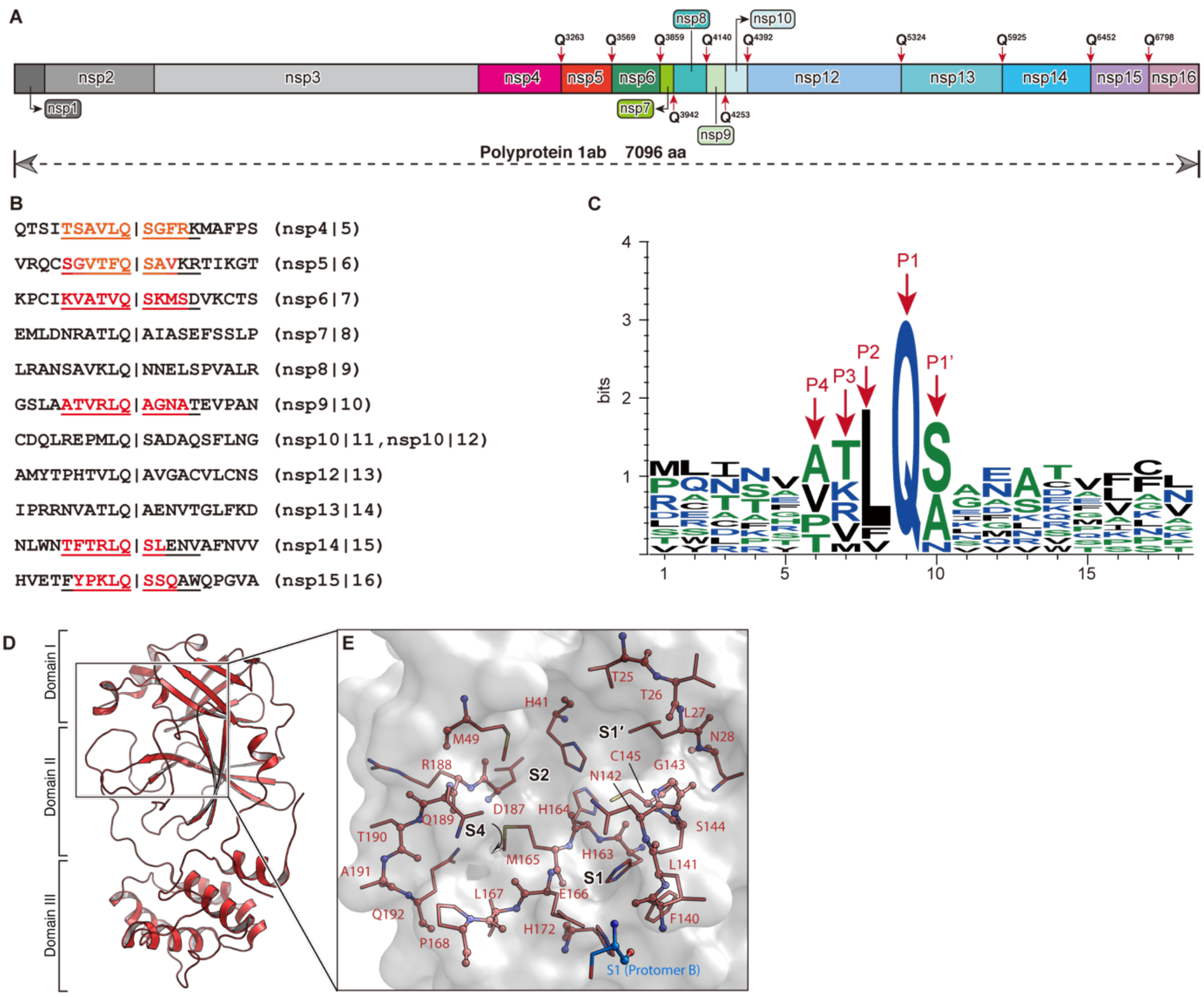

Fig. S1. Residues participate in the formation of SARS-CoV-2 M^pro^ substrate binding pocket and its peptidyl substrate. (A) The schematic diagram of pp1ab. The colored arrow represents M^pro^ cleavage site. Conserved glutamine residues are labeled. **(B)** Residues around 11 cleavage sites of M^pro^. The underlined residues represent synthesized peptide for structural and functional study in this paper. And the red colored residues represent residues that can be traced in the peptidyl substrate complex. **(C)** Sequence alignment of residues around 11 cleavage sites. The size of letters indicates the conservative property. **(D)** The overall structure of M^pro^. **(E)** The zoom-in view of the substrate binding pocket. Residues that participate in the formation of substrate binding site are shown in ball-and-stick model.

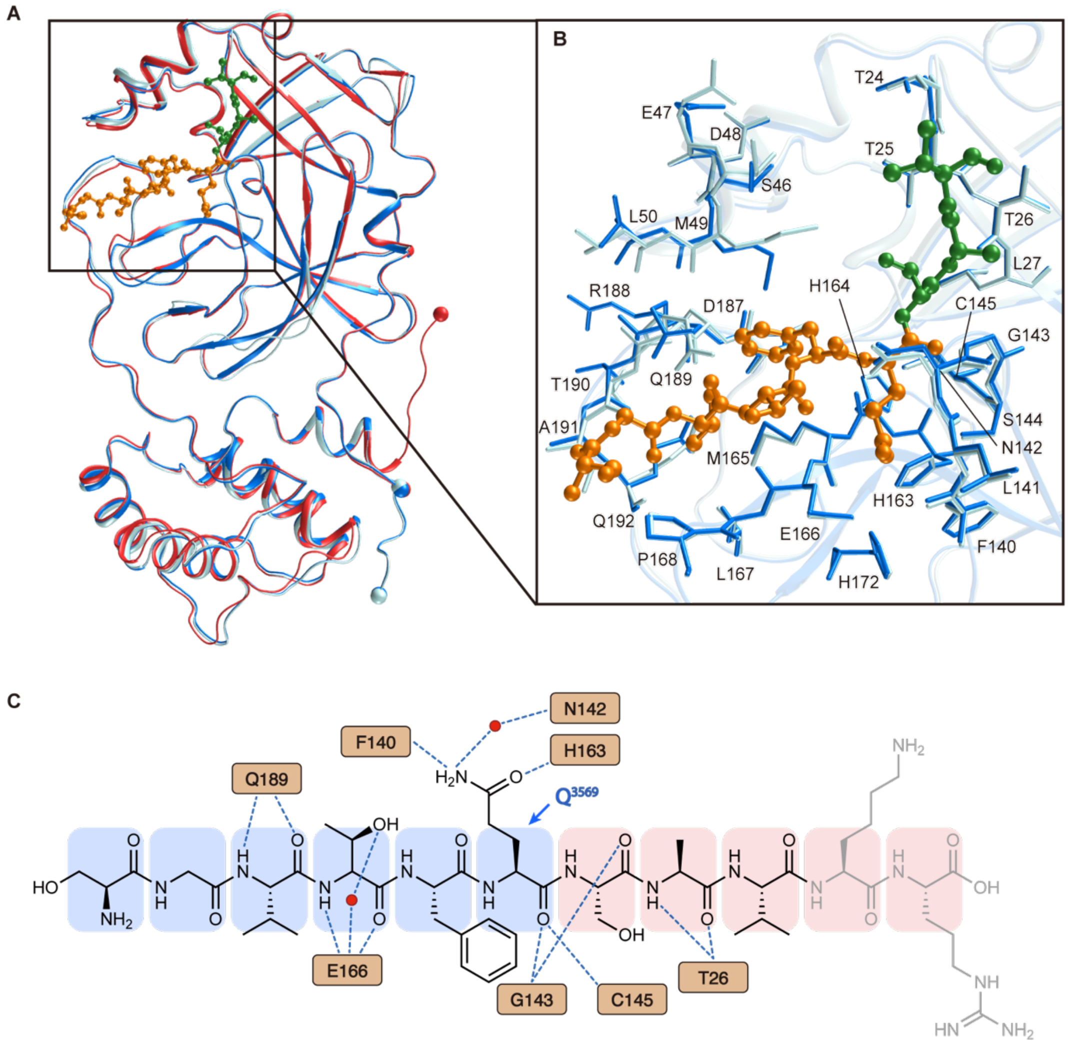

Fig. S2. Interactions between H41A mutant and nsp5|6. (A) The comparison of M^{pro} (deep salmon), H41A mutant (cyan) and H41A-nsp5|6 structure (marine). **(B)** The zoom-in view of substrate binding pocket. Residues that participated in binding pocket formation are shown as sticks. Peptidyl substrate shows in ball-and-stick model. Residues from P1-P6 and P1′-P3′ are colored in orange and green, respectively. **(C)** The network of hydrogen bonds between H41A mutant and nsp5|6. Residues that can be traced according to the electron density map are colored in black. Residues that cannot be traced are colored in gray. Residues that form hydrogen bonds with nsp5|6 are represented in orange square. Water molecules are shown as red spheres. Q^{3569} is highlighted with a blue arrow. Background of P1 to P6 residues is colored blue while background of P1′ to P5′ is colored pink.

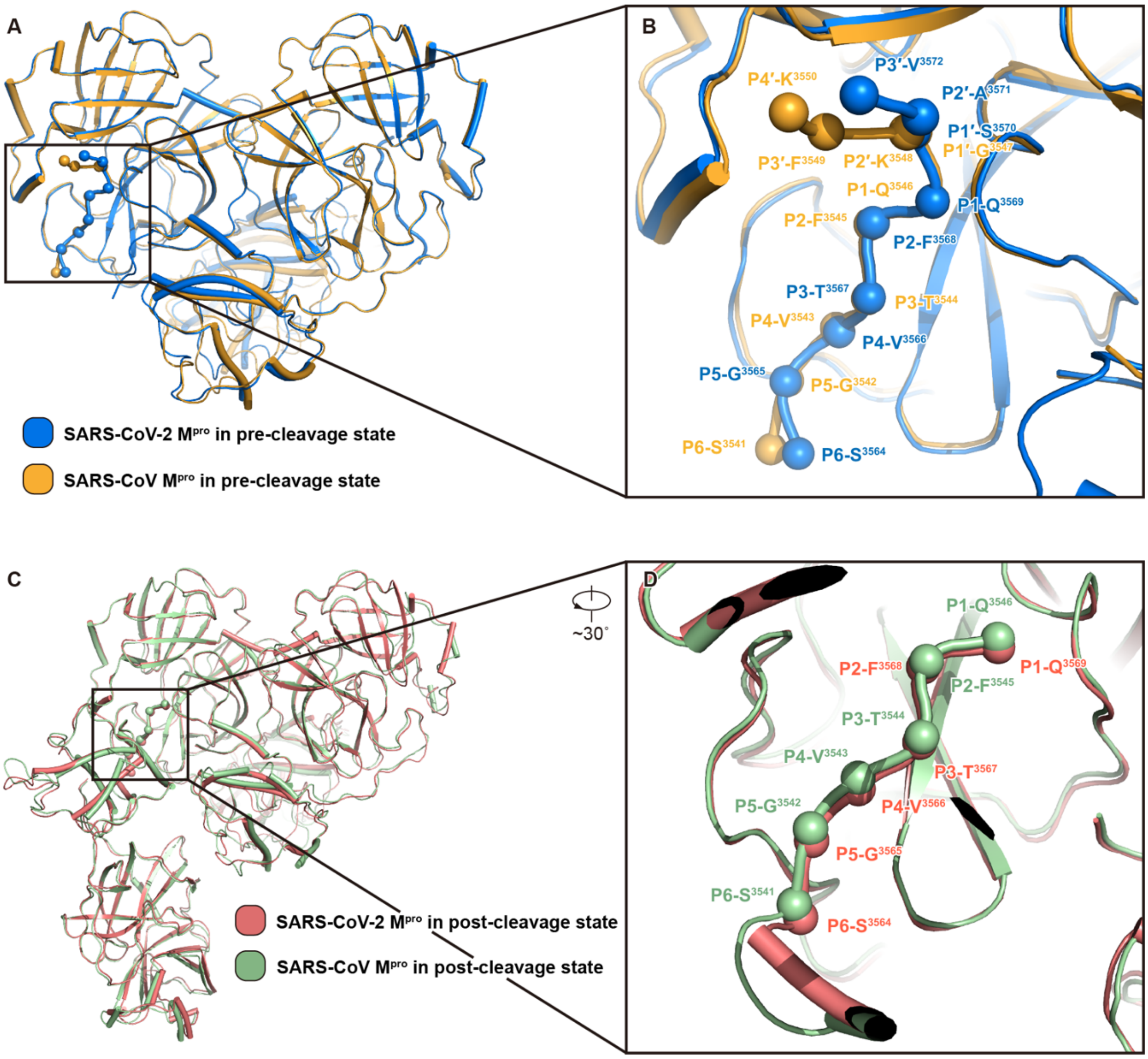

Fig. S3. Comparison of SARS-CoV-2 M^{pro} and SARS-CoV M^{pro} in post-cleavage state and pre-cleavage state. (A) Superposition of SARS-CoV-2 M^{pro} in pre-cleavage state (H41A-nsp5|6 complex) (PDB code: 7DVW) with SARS-CoV M^{pro} in pre-cleavage state (PDB code: 5B6O). SARS-CoV-2 M^{pro} in pre-cleavage state is shown in blue. SARS-CoV M^{pro} in pre-cleavage state is shown in orange. **(B)** The zoom-in view of the substrate binding pocket from (A). Each Cα of substrates is shown as a colored sphere. **(C)** Superposition of SARS-CoV-2 M^{pro} in post-cleavage state (PDB code: 7E5X) with SARS-CoV M^{pro} in post-cleavage state (PDB code: 1Z1J). SARS-CoV-2 M^{pro} in post-cleavage state is shown in salmon. SARS-CoV M^{pro} in post-cleavage state is shown in green. **(D)** The zoom-in view of the substrate binding pocket from (**C**). Each Cα of P6 to P1 positions from substrates is shown as a colored sphere.

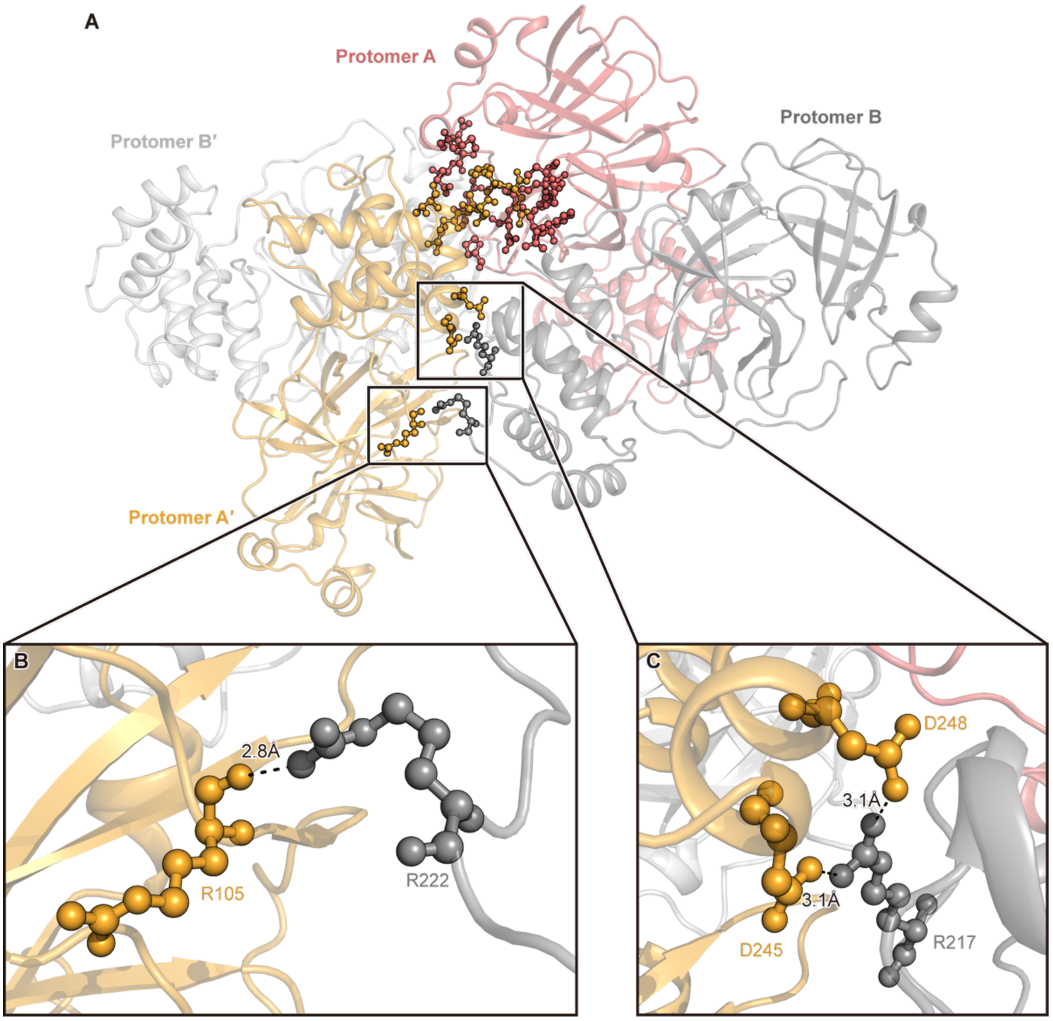

Fig. S4. Extra interactions between SARS-CoV-2 M^{pro} and its natural protein substrate. (A) Extra hydrogen interactions beyond substrate binding pocket. Zoom-in view of two different sites are shown in **(B)** and **(C)**.

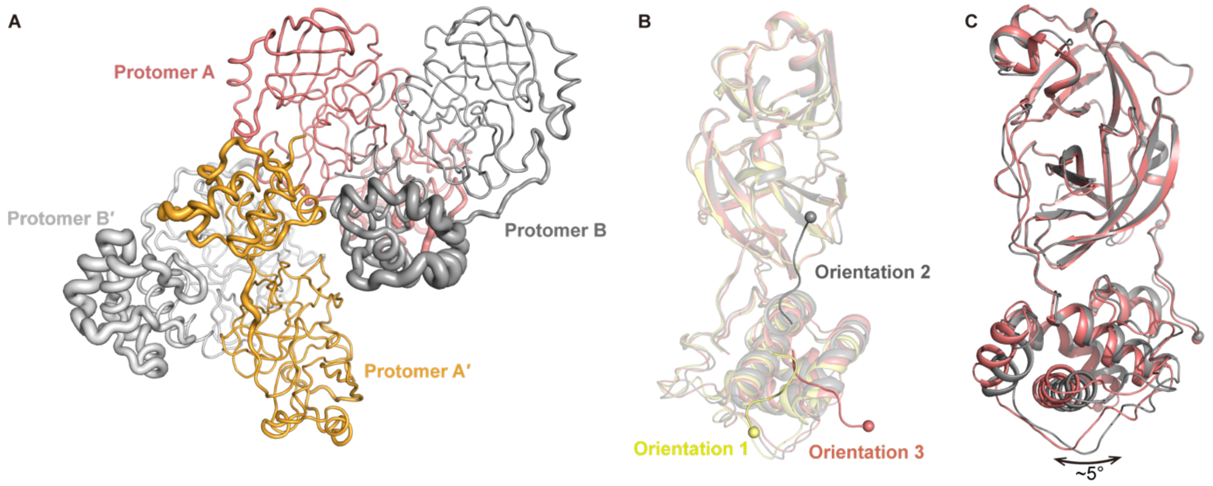

Fig. S5. The conformation diversity of protomers in the structure of SARS-CoV-2 M^{pro} in complex with its natural protein substrate. (A) Structure of SARS-CoV-2 M^{pro} in complex with its natural protein substrate represented through B factor values. **(B)** The comparison of protomer A (deep salmon), protomer B (dark gray) and H41A mutant (yellow). Residues from 301-306 are highlighted. The Cα of Q306 is represented as spheres. Orientation 1, orientation 2, and orientation 3 are labeled. **(C)** The comparison of protomer A (deep salmon) and protomer B (dark gray) from the AB dimer.

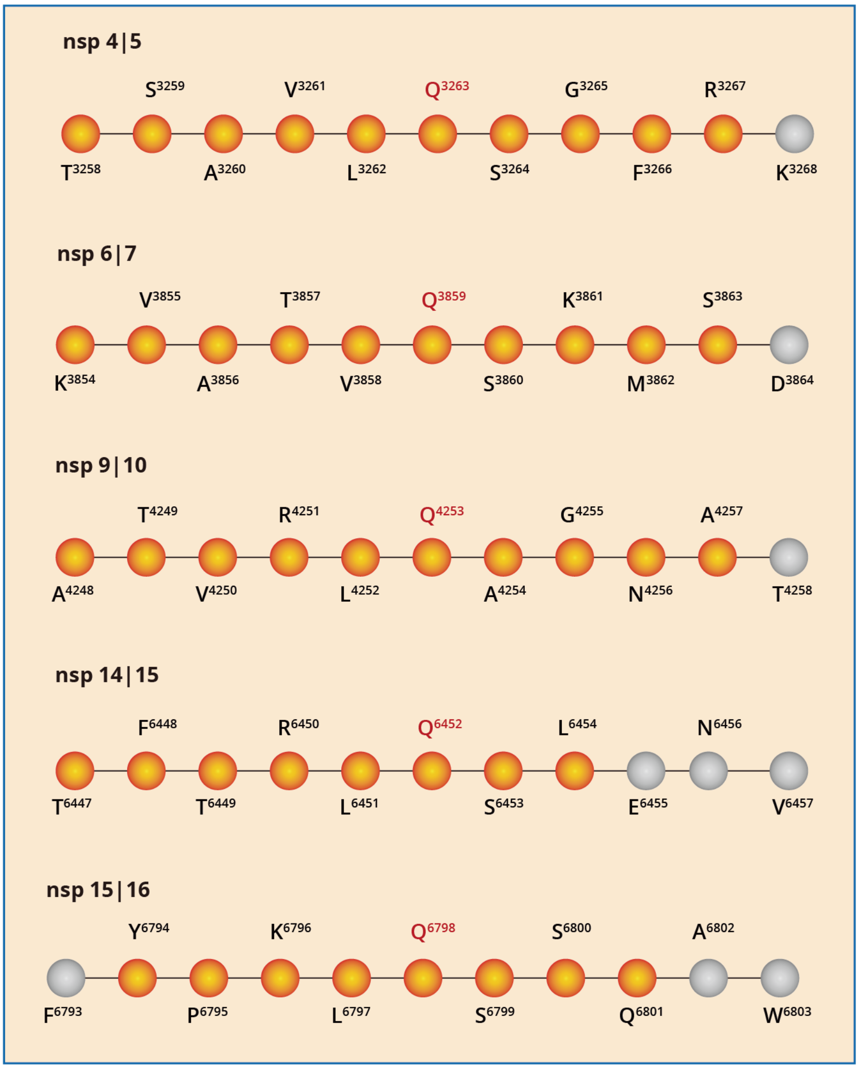

Fig. S6. The schematic diagram of nsp4|5, nsp6|7, nsp9|10, nsp14|15 and nsp15|16. Residues that can be traced according to the electron density map are colored in orange. Residues that cannot be traced are colored in gray.

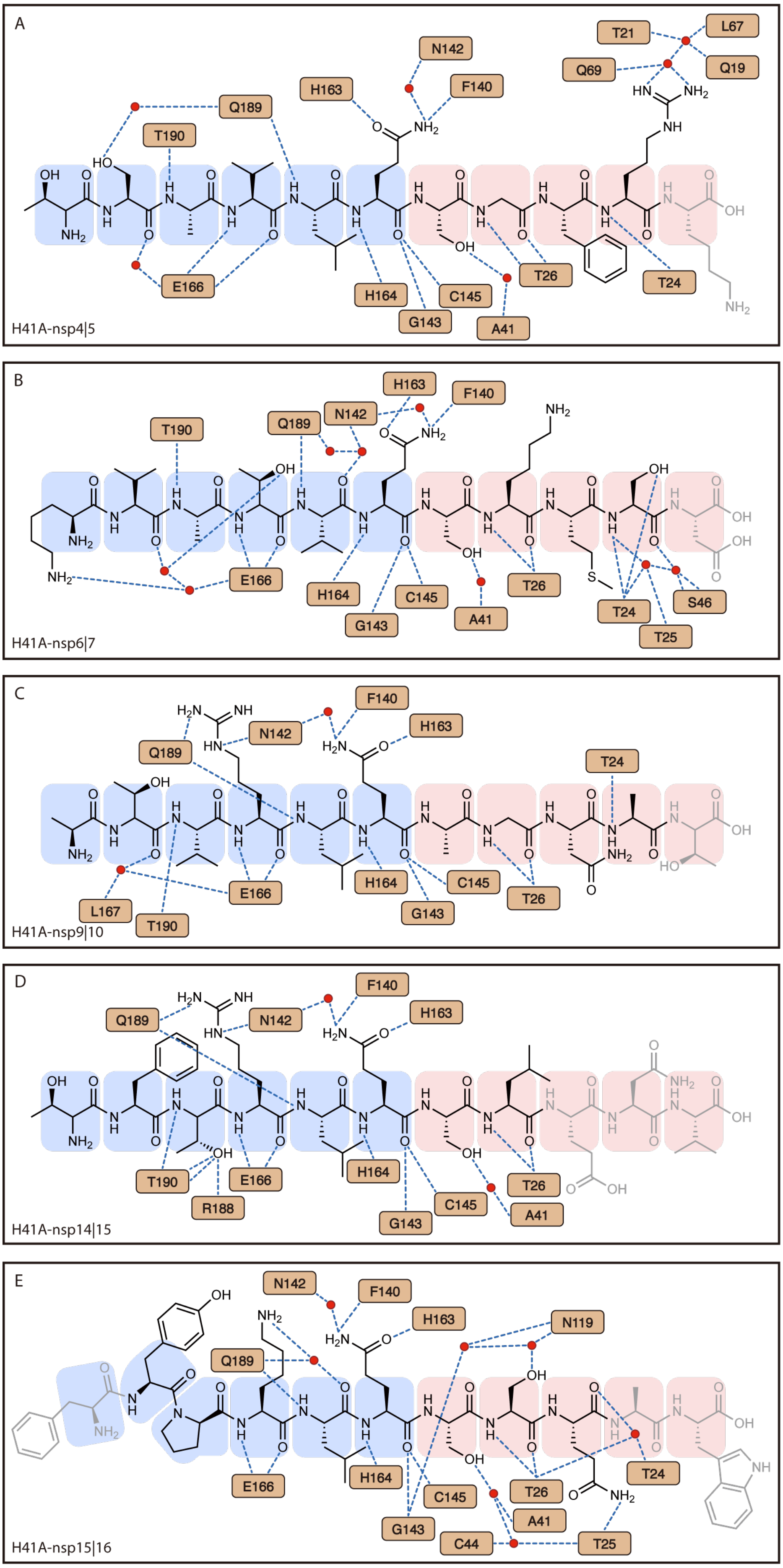
A
T21
L67
Q69
Q19
N142
H163
F140
Q189
T190
E166
H164
C145
G143
A41
T26
T24
H41A-nsp4|5
B
H163
F140
N142
Q189
T190
E166
H164
C145
G143
A41
T26
T24
T25
S46
H41A-nsp6|7
C
F140
N142
H163
Q189
T24
E166
H164
C145
T26
G143
L167
T190
H41A-nsp9|10
D
F140
Q189
N142
H163
T190
E166
H164
T26
R188
C145
A41
G143
H41A-nsp14|15
E
N142
F140
H163
N119
Q189
E166
H164
C145
T26
T24
A41
G143
C44
T25
H41A-nsp15|16

Fig. S7. The hydrogen bonds networks between SARS-CoV-2 M^{pro} and its peptidyl substrates. (A) H41A-nsp4|5, **(B)** H41A-nsp6|7, **(C)** H41A-nsp9|10, **(D)** H41A-nsp14|15, **(E)** H41A-nsp15|16. The interaction network of hydrogen bonds between H41A and peptidyl substrates (nsp4|5, nsp6|7, nsp9|10, nsp14|15 and nsp15|16). Residues that can be traced according to the electron density map are colored in black. Residues that can't be traced are colored in gray. Residues that form hydrogen bonds with peptidyl substrates are highlighted with orange squares. Water molecules are shown as red spheres. Background of P1 to P6 residues is colored purple while background of P1′ to P5′ is colored pink.

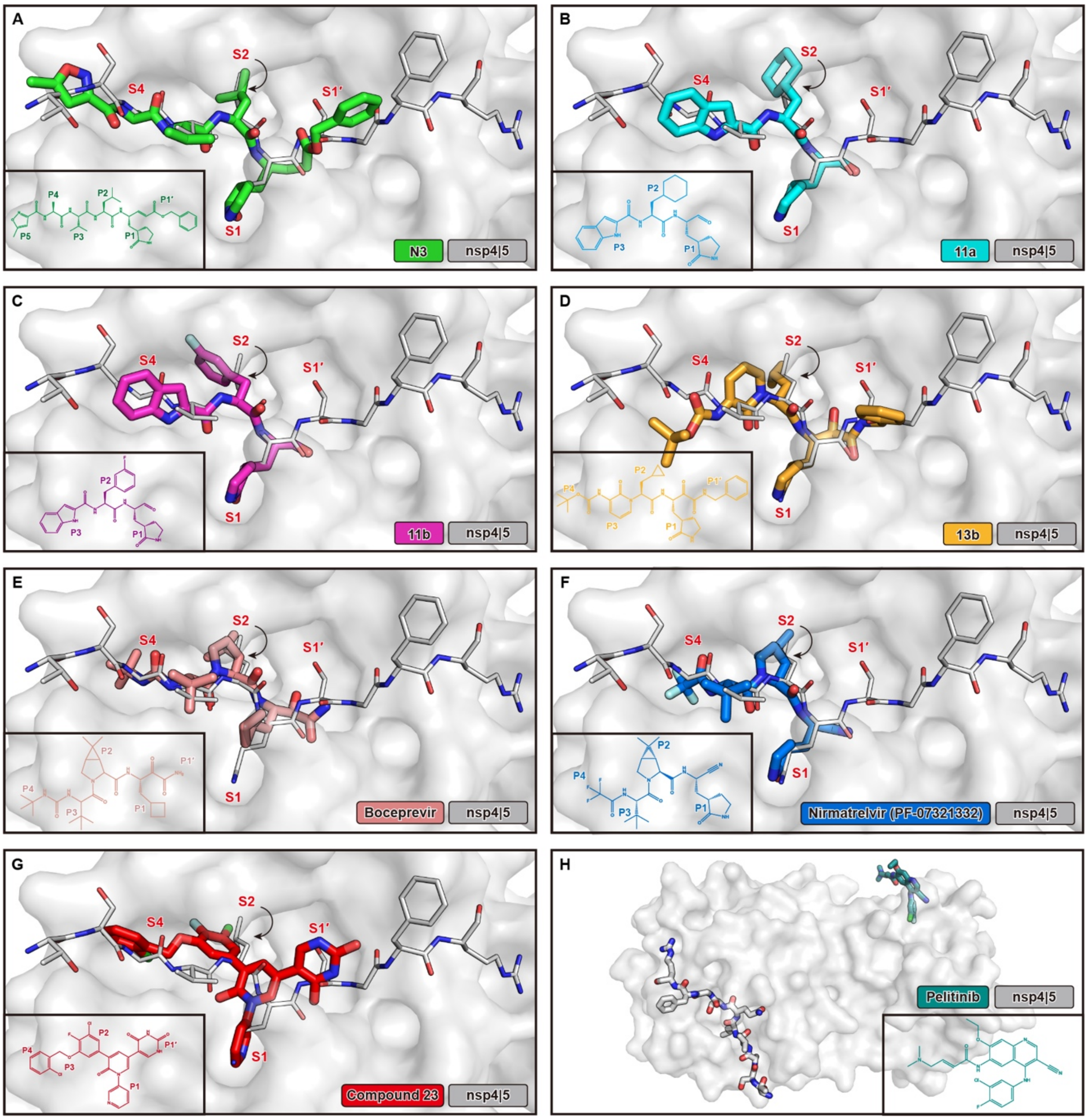

Fig. S8. The comparison of binding model between selected M^{pro} inhibitors and M^{pro} cleavage substrate nsp4|5. Comparison of binding modes of H41A-nsp4|5 (PDB code: 7DVP) to **(A)** M^{pro}-N3 (PDB code: 6LU7), **(B)** M^{pro}-11a (PDB code: 6LZE), **(C)** M^{pro}-11b (PDB code: 6M0K), **(D)** M^{pro}-13b (PDB code: 6Y2F), **(E)** M^{pro}-boceprevir (PDB code: 7C6S), **(F)** M^{pro}-nirmatrelvir (PF-07321332) (PDB code: 7VH8), **(G)** M^{pro}-compound 23 (PDB code: 7M8P), and **(H)** M^{pro}-pelitinib (PDB code: 7AXM).

Table S1. Data collection and refinement statistics.

	M^{pro}-Apo	H41A	M^{pro}-Post-cleavage state
	PDB code: 6M03	PDB code: 7VAH	PDB code: 7E5X
**Data Collection**			
Space group	*C2*	*C2*	*P1*
Wavelength (Å)	0.9793	0.9785	0.9789
Cell dimensions			
a, b, c (Å)	113.964, 53.450, 45.029	98.201, 83.048, 51.597	63.48, 73.78, 73.79
α, β, γ (°)	90, 101.83, 90	90, 115.58, 90	66.40,90.01,90,01
Resolution (Å)	50.00-1.99 (2.04-1.99)	50.00-1.49 (1.53-1.49)	46.33-2.19 (2.25-2.19)
No. of unique reflections	35505 (2589)	60133 (4053)	61149 (4382)
Completeness (%)	99.5 (97.8)	98.8 (90.3)	97.1 (94.6)
R_{merge} (%)[a]	3.9 (62.5)	3.7 (91.5)	3.7 (109.4)
Mean $I/\sigma I$	16.29 (1.87)	21.62 (2.09)	16.94(1.18)
*CC*1/2	99.9 (75.2)	100.0 (80.0)	99.9(63.6)
Redundancy	3.47 (3.48)	6.59 (6.46)	3.54(3.63)
Wilson B factors (Å^2)	41.54	31.46	59.4
**Refinement**			
Resolution (Å)	38.65-2.0	42.54-1.49	46.33-2.19
No. of reflections used		60102	58081
R_{work} / R_{free} (%)	19.67/24.77	19.32/21.66	23.47/26.61
No. atoms			
Protein	2367	2381	9318
Ligand/ion			
Water	87	359	80
B-factors (Å^2)			
Protein	52.28	32.9	83.89
Ligand/ion			
Water	54.88	44.09	80.58
R.m.s. deviations			
Bond lengths (Å)	0.003	0.003	0.012
Bond angles (°)	0.72	0.621	1.7
Ramachandran plot (%)			
Favored (%)	96.71	99.01	92.27
Allowed (%)	3.29	0.99	7.73
Outliers (%)	0	0	0

(Continued)

	H41A-nsp4|5	H41A-nsp5|6	H41A-nsp6|7
	PDB code: 7DVP	PDB code: 7DVW	PDB code: 7DVX
**Data Collection**			
Space group	*C2*	*C2*	*C2*
Wavelength (Å)	0.9785	0.9785	0.9785
Cell dimensions			
a, b, c (Å)	98.228, 82.581, 54.145	96.390, 81.976, 54.386	98.626, 82.546, 51.729
α, β, γ (°)	90, 117.79, 90	90, 117.17, 90	90, 115.48, 90
Resolution (Å)	50.00-1.69 (1.73-1.69)	20.00-1.49 (1.53-1.49)	50.00-1.80 (1.84-1.80)
No. of unique reflections	73760 (6011)	59894(3579)	67652 (4992)
Completeness (%)	87.2 (96.1)	97.8 (79.4)	98.3 (98.4)
R_{merge} (%)[a]	5.1 (44.0)	4.9 (69.3)	5.6 (54.8)
Mean $I/\sigma I$	13.71 (2.28)	19.72 (2.16)	13.78 (2.22)
*CC*1/2	99.9 (88.3)	99.9 (90.2)	99.8 (79.8)
Redundancy	3.28 (3.32)	6.61 (4.66)	3.41 (3.34)
Wilson B factors ($Å^2$)	29.69	30.29	32.25
**Refinement**			
Resolution (Å)	43.96-1.69	19.75-1.49	46.70-1.80
No. of reflections used	37823	59816	34602
R_{work} / R_{free} (%)	17.74/21.61	16.39/18.83	19.94/24.72
No. atoms			
Protein	2365	2368	2377
Ligand/ion	74	66	73
Water	319	293	301
B-factors ($Å^2$)			
Protein	28.61	35.9	30.77
Ligand/ion	36.65	49.18	39.06
Water	38.72	48.76	39.92
R.m.s. deviations			
Bond lengths (Å)	0.006	0.017	0.004
Bond angles (°)	0.783	1.447	0.785
Ramachandran plot (%)			
Favored (%)	97.76	98.07	96.79
Allowed (%)	1.92	1.93	3.21
Outliers (%)	0.32	0	0

(Continued)

	H41A-nsp9|10	H41A-nsp14|15	H41A-nsp15|16
	PDB code: 7DVY	PDB code: 7DW6	PDB code: 7DW0
**Data Collection**			
Space group	*C2*	*C2*	*C2*
Wavelength (Å)	0.9785	0.9785	0.9785
Cell dimensions			
a, b, c (Å)	98.670, 82.048, 51.738	97.790, 80.980, 51.710	96.723, 81.660, 54.386
α, β, γ (°)	90, 115.46, 90	90, 114.60, 90	90, 117.33, 90
Resolution (Å)	50.00-1.80 (1.84-1.80)	30.68-1.81(1.86-1.81)	50.00-1.70 (1.74-1.70)
No. of unique reflections	65423 (4829)	33373 (2428)	78769 (5635)
Completeness (%)	95.3 (95.1)	99.7 (99.6)	95.8 (93.8)
R_{merge} (%)[a]	6.8 (51.9)	5.8 (87.0)	4.0 (62.4)
Mean $I/\sigma I$	10.91 (2.32)	15.4 (2.3)	16.15 (1.96)
*CC*1/2	99.7 (79.8)	99.9 (79.9)	99.9 (82.3)
Redundancy	3.54 (3.61)	6.8 (6.9)	3.52 (3.53)
Wilson B factors (Å^2)	32.3	42.98	34.05
**Refinement**			
Resolution (Å)	46.71-1.80	25.83-1.81	48.14-1.70
No. of reflections used	33472	33369	40192
R_{work} / R_{free} (%)	17.51/21.63	17.45/20.77	18.77/21.83
No. atoms			
Protein	2334	2321	2362
Ligand/ion	69	65	66
Water	316	199	279
B-factors (Å^2)			
Protein	28.56	41.65	36.04
Ligand/ion	35.56	68.12	46.76
Water	39.46	50.24	44.75
R.m.s. deviations			
Bond lengths (Å)	0.008	0.009	0.006
Bond angles (°)	0.921	0.941	0.742
Ramachandran plot (%)			
Favored (%)	98.7	97.37	96.77
Allowed (%)	1.3	2.63	3.23
Outliers (%)	0	0	0
